# Secondhand smoke exposure in public outdoor spaces in the Netherlands: The stronger the smell, the more exposure to nicotine

**DOI:** 10.18332/tid/186952

**Published:** 2024-05-17

**Authors:** Jeroen Bommelé, Hans Cremers, Wouter Den Hollander, Sigrid Troelstra, Gemma Geuke, Wiebe Dam, Eefje Willemse, Petra Hopman, Bethany Hipple Walters, Marc Willemsen

**Affiliations:** 1 The Netherlands Expertise Centre for Tobacco Control, Trimbos Institute, Utrecht, The Netherlands; 2 National Institute for Public Health and the Environment, Bilthoven, Τhe Netherlands; 3 Department of Health Promotion, Maastricht University, Maastricht, Τhe Netherlands

**Keywords:** smoking, nicotine, 3-EP, secondhand smoke, smoke-free policies

## Abstract

**INTRODUCTION:**

While secondhand smoke exposure in outdoor spaces has been investigated before, no data on outdoor secondhand smoke exposure have been collected in the Netherlands. Such data could help policymakers gain support for smoke-free outdoor public spaces.

**METHODS:**

Between May and November 2021, we visited 25 outdoor locations across the Netherlands. At each location, we conducted four measurements with smokers and one measurement without smokers. During each measurement, we counted the number of smokers present and we rated tobacco smell intensity on a five-point scale. Airborne nicotine and 3-ethenylpyridine (3-EP) data were collected through active sampling on thermal desorption tubes. The contents of these tubes were later analyzed using gas chromatography-mass spectrometry. Using linear mixed models, we investigated the association between levels of nicotine and the presence of smokers, the number of smokers, and the intensity of tobacco smell. We also investigated these association with levels of 3-EP.

**RESULTS:**

Nicotine levels were higher when smokers were present (B=1.40; 95% CI: 0.69–2.11, p<0.001). For each additional smoker present, we measured higher levels of nicotine (B=0.23; 95% CI: 0.10–0.37, p=0.001). When the smell of tobacco smoke was noted to be stronger by the researchers, higher levels of nicotine were measured through sampling (B=0.85; 95% CI: 0.44–1.26, p<0.001). We found similar results for 3-EP levels.

**CONCLUSIONS:**

This study showed that both nicotine and 3-EP are useful in quantifying levels of secondhand smoke in various outdoor locations. The level of nicotine exposure outdoors was positively associated with the number of smokers nearby. The intensity of the tobacco smell was also related to nicotine exposure: the stronger the smell of tobacco smoke, the more nicotine was measured in the air.

## INTRODUCTION

About 1.5 to 1.9 billion non-smokers are exposed to secondhand smoke worldwide^1^, causing major health problems in those non-smokers^2^. Moreover, an estimated 1.3 million people die prematurely each year due to long-term secondhand smoke exposure^3^, and many others suffer from diseases such as ischemic heart disease and asthma^4^. While long-term exposure to secondhand smoke causes death and illness, short-term exposure also has health consequences. For example, after one hour of exposure at levels common at café terraces, a person may experience inflammatory reactions and a significantly decreased lung function^5^. Comprehensive smoke-free policies are an effective way of protecting non-smokers from these long-term and short-term health effects of secondhand smoke exposure^1^.

### Smoke-free policies in the Netherlands

In the past decades, the government of the Netherlands implemented several national smoke-free policies^6^. The first smoke-free policies focused on indoor settings: smoking was banned in public buildings (1990), indoor workplaces (2004), public transport (2008), and hospitality settings (partially in 2008, fully in 2014)^6^. Inspired by the Dutch Movement Towards a Smoke-free Generation^7^, a growing number of public and private outdoor spaces have voluntarily become smoke-free in the Netherlands since 2017 and to the present^8^. This movement aims to create a society in which all children born after 2017 are able to grow up smoke-free and without exposure to tobacco smoke. The government of the Netherlands also legally banned smoking on school grounds (2020), while owners of various other outdoor settings frequently visited by children, such as playgrounds and sports grounds, continued to voluntarily create smoke-free spaces on their grounds^7^. In addition, a growing number of childcare facilities, hospitals, universities, and private businesses have made their campuses smoke-free between 2020 and 2023^9^. Despite these efforts, many outdoor spaces where smoking is allowed exist in the Netherlands and non-smokers continue to be exposed in these outdoor spaces. Clear statistics on secondhand smoke exposure levels may help national and local policy makers and tobacco control advocacy groups to raise awareness of secondhand tobacco smoke exposure.

### Measuring secondhand smoke exposure

There are various methods for measuring and substantiating exposure to secondhand smoke. Fine particulate matter (PM_2.5_) is the most commonly used indicator as it is relatively easy to measure and requires no laboratory analyses^10,11^. Airborne nicotine and 3ethenylpyridine (3-EP) are also well-established indicators of secondhand smoke exposure but testing for these substances generally requires laboratory analyses^11,12^. One advantage of nicotine and 3-EP over fine particulate matter, however, is that nicotine directly indicates exposure to both traditional tobacco products (cigarettes) or non-traditional sources (e.g. e-cigarettes and heated tobacco products)^11^. Another advantage of nicotine is that fine particulate matter may have other sources than tobacco smoke, such as car exhausts and smoke from fireplaces. Fine particulate matter therefore corresponds to a less extent to secondhand smoke exposure.

While numerous studies have investigated secondhand smoke exposure in outdoor spaces before^10-12^, no such data have been collected in the Netherlands. Also, while many studies used PM_2.5_ as their main indicator for outdoor secondhand smoke exposure, few used nicotine and 3-EP as indicators^10,12^. One notable example is the study of Fu et al.^12^ in which the authors used an air sampling pump and glass fiber filters concealed in a bag to measure airborne nicotine levels inside and around cafés and restaurants in Barcelona; on 51 outdoor cafés and terraces, they found a median level of nicotine of 0.54 μg/m^3^. Interestingly, the researchers were not only able to show that these levels were higher when more smokers were present, but also when the researchers smelled tobacco smoke.

In the present study, we investigated secondhand smoke exposure levels at 25 locations across three types of public outdoor settings in the Netherlands. We measured nicotine and 3-EP levels at main public transport stops, on café terraces, and at public building entrances. We chose these particular outdoor settings as they share characteristics with most other open, semi-open, and enclosed outdoor locations that have not yet been made smoke-free. In addition to assessing nicotine and 3-EP levels, we also investigated whether the number of smokers present and the level of tobacco smell were associated with higher levels of secondhand smoke exposure.

## METHODS

### Study design and locations

Between May and November 2021, we visited 25 outdoor locations across the Netherlands. These locations included public transport stops (primarily the central bus station), café terraces, and entrances of public buildings. We aimed to collect data when different numbers of smokers were present and across various tobacco smell intensity levels. Therefore, at each location, we planned to conduct four measurements with smokers and one measurement without smokers (i.e. baseline measurement). We deviated slightly from this protocol at five locations (Supplementary file Table S1), due to practical challenges (e.g. smokers entering a location during a baseline measurement). Our study design allowed for such deviations because we were only interested in the overall variations in the number of smokers and tobacco smell intensity levels. In total, at the 25 locations, we conducted 97 measurements with smokers and 25 baseline measurements without smokers (Supplementary file Table S1).

### Procedure

At each location, two researchers conducted the data collection. One researcher collected nicotine and fine particle data using a backpack with a SidePak^TM^ AM520 personal aerosol device and a GilAir^®^ Plus personal air sampling pump inside. Two rubber tubes were connected on one end to one of the devices inside the backpack, and to the backpack’s shoulder straps on the other end. This way, both devices were hidden from view while enabling the researcher to sample air from outside the backpack. The intake of the devices was located at approximately the same height as the mouth and nose of the person carrying the device. As both devices produced significant levels of noise, we used soundproofing material inside the backpack to avoid detection by bystanders. Unfortunately, due to an equipment malfunction, we were unable to use the fine particle data from the a SidePak^TM^ AM520. We elaborate on this in the Discussion section. During each measurement, the researcher wearing the backpack (JB), recorded data on all smokers nearby (within 10 m). A second researcher (ST, GG, WD, or EW) used a mobile device to record location characteristics, tobacco smell intensity, and weather conditions.

We visited three types of locations: entrances of public buildings (n=7), public transport stops (n=9), and café terraces (n=8). All measurements were conducted between 8:00 and 17:00 on days with mostly favorable weather conditions (i.e. no rain, relative humidity <85%). Within this time bracket, we identified suitable locations and began collecting data whenever smokers in the area started to smoke or were about to smoke. If we were unable to conduct a baseline measurement at a location (i.e. one without smokers), we used a location with similar characteristics nearby at >10 m from the original location^13^.

### Building entrances

Measurements were taken just outside the entrance of public buildings, such as train stations, government buildings, and shopping malls.

### Public transport stops

Data collection at public transport stops was similar to that at public entrances. We visited large bus stops near train stations to increase the chance of finding a suitable location. At each location, we positioned ourselves in the center of the bus shelter (if present) to represent the most typical location travelers would be waiting for their bus.

### Café terraces

Upon arrival, we looked for patrons sitting in the outdoor dining area who either were smoking or had a pack of cigarettes on their table. The measurements at this location were guided by the work done by Cameron et al.^14^. We then positioned ourselves at a table nearby, placing the backpack on a chair next to the researchers. This way, the exposure measured by the backpack resembled real-life exposure by non-smoking patrons. One challenge at café terraces was that the measurements took place during the COVID-19 pandemic. In the data collection period, hospitality venues were required to impose a minimum distance of 1.5 m between tables and patrons. It is likely that smokers and non-smokers alike were more aware of their physical distance with regard to other people and were more likely to keep a greater distance from others, including distance from members of the research team. This may have somewhat influenced the sampling.

### Measurements and variables

*Nicotine and 3-EP*

Airborne nicotine and 3-EP (3ethenylpyridine) were collected through active sampling on thermal desorption tubes (TD-tubes). The tubes were attached to a GilAir^®^ Plus personal air sampling pump set at a flow rate of 400 cc per minute for ten minutes. At four locations, the flow rate was set at 300 cc per minute (Supplementary file Table S1). The thermal absorption tubes were later analyzed in the laboratory by the second author (HC) using gas chromatography-mass spectrometry. Details on these analyses and the laboratory settings used can be found in Supplementary file Part 1.

*Number of smokers*

At the start of each measurement, we counted the number of smokers within 10 m of the backpack. Whenever someone started smoking within a range of 10 m during the measurement, we recorded their time of starting smoking, distance, and relative location to the equipment, relative wind direction, and type of tobacco product used (i.e. smoking cigarettes, cigars, or electronic cigarettes). We documented these data using an online data collection form.

*Tobacco smell intensity*

One researcher subjectively rated the intensity of the tobacco smell at the start of each measurement, halfway into the measurement (after 5 min), and at the end of the measurement (after 10 min). We used the average of these three scores to calculate an overall tobacco smell intensity score per measurement. The smell was subjectively categorized as: ‘no smell’, ‘light smell’, ‘moderate smell’, ‘strong smell’, and ‘predominant smell’ (score 0–4). Even though smell is a highly subjective measurement, we aimed to create a more objective measurement by giving researchers instructions for specific levels of smell intensity. ‘No smell’ was to be reported when the researcher smelled absolutely no tobacco smoke. ‘Light smell’ was a faint smell, which one would barely detect had one not paid attention to it. A ‘moderate smell’ was a smell that was clearly detectable, but still bearable. A ‘strong smell’ was one that one that could be considered annoying and a ‘predominant smell’ would make one want to leave the area.

*Weather conditions*

Temperature and humidity are known technical confounders when measuring nicotine and 3-EP^15^. However, due to the nature of our data collection, we were unable to bring equipment for measuring local temperature and humidity levels. Instead, an online weather forecast website for estimating local temperature and humidity levels was used (weather.com). An overview of the weather conditions per measurement can be found in Supplementary file Table S1.

### Statistical analysis

First, we calculated the overall mean and median levels of nicotine and 3-EP. Next, we created three linear mixed models to investigate the association between levels of nicotine and: 1) the presence of smokers, 2) the number of smokers; and 3) the intensity of tobacco smell. In the first model, we used condition (smokers present: yes vs no) as an independent variable of interest. In the second model, we used number of smokers (range 0–14), and in the third model we used tobacco smell intensity (scale 0–4), as independent variables of interest. Location was added as a random intercept and the models were further adjusted by including local temperature and humidity as fixed effects. After creating each model, we visually inspected the residual values for a normal distribution. Finally, the same analyses were performed utilizing 3-EP as the dependent variable. We used SPSS 29.0 for all analyses and chose a level of significance of p<0.05 for those analyses. Where applicable, all tests were two-tailed.

### Ethical considerations

The Central Committee on Research Involving Human Subjects in the Netherlands requires no ethical approval for research without human subjects. No personally identifiable data were collected on individuals at any of the locations.

Before conducting this study, we considered that the researchers in this study would be exposed to potentially high levels of secondhand tobacco smoke during the data collection. All data-collecting researchers had therefore been given a free choice to participate in the study and all consented to do so. During the entire data collection, it was the first author – who conceived the study – who wore the backpack with equipment and was thus most exposed to tobacco smoke. To monitor the individual exposure levels of researchers, we measured exhaled carbon monoxide levels before and after each location. These levels never exceed 2 ppm or showed any increase during data collection, suggesting that researchers had been exposed to very low levels of tobacco smoke.

## RESULTS

Table 1 presents mean and median nicotine and 3-EP levels per location type. Supplementary file Table S1 presents the number of measurements, weather conditions, and flow rate used for the thermal desorption tubes (nicotine and 3-EP measurements).

**Table 1:** Mean and median levels (μg/m^3^) of secondhand smoke indicators at public outdoor spaces in the Netherlands in 2021

	*No smokers*			*With smokers*		
	*n*	*Mean*	*Median*	*n*	*Mean*	*Median*
**All locations**						
Nicotine	25	0.414	0.320	97	1.793	1.330
3-EP	25	0.077	0.000	97	0.237	0.220
**Building entrances**						
Nicotine	7	0.581	0.380	28	1.789	1.745
3-EP	7	0.083	0.000	28	0.234	0.240
**Public transport stops**						
Nicotine	10	0.369	0.340	35	2.356	1.560
3-EP	10	0.065	0.000	35	0.295	0.230
**Café terraces**						
Nicotine	8	0.323	0.310	34	1.216	0.480
3-EP	8	0.088	0.000	34	0.179	0.095

Nicotine and 3-EP levels are presented in microgram per cubic meter. n: number of measurements (about 5 per location: 1 without smokers and 4 with smokers).

### Nicotine

We calculated three separate models for predicting nicotine. The results of these models are presented in Table 2. Mean nicotine levels were higher when smokers were present than when they were not (B=1.40; 95% CI: 0.69–2.11, p<0.001). Also, mean nicotine levels were higher when more smokers were present (B=0.23; 95% CI: 0.10–0.37, p=0.001). Finally, mean nicotine levels were higher when the smell of tobacco smoke was stronger (B=0.85; 95% CI: 0.44–1.26, p<0.001).

**Table 2:** Mixed linear models for predicting nicotine level at public outdoor spaces in the Netherlands in 2021

	*B*	*SE B*	*df*	*t*	*p*
**Condition**					
Constant	-1.78	2.33	43	-0.76	0.450
Condition	1.40	0.36	95	3.91	**<0.001**
Temperature	0.00	0.06	35	-0.07	0.948
Humidity	0.03	0.02	41	1.72	0.094
**Number of smokers**					
Constant	-2.36	2.40	46	-0.98	0.330
Number of smokers	0.23	0.07	108	3.38	**0.001**
Temperature	0.03	0.07	37	0.49	0.625
Humidity	0.04	0.02	43	2.05	**0.047**
**Tobacco smell**					
Constant	-1.93	2.36	43	-0.82	0.417
Tobacco smell	0.85	0.21	106	4.13	**<0.001**
Temperature	0.03	0.07	36	0.46	0.648
Humidity	0.03	0.02	41	1.57	0.123

The table presents three separate mixed linear models for condition (no smokers vs smokers, reference: no smokers), number of smokers (range: 0–14), and tobacco smell (range: 1–4). We conducted 5 measurements per location. To control for this, location was added as a random effect. Significance: p<0.05.

### 3-EP

**Figure 1:**
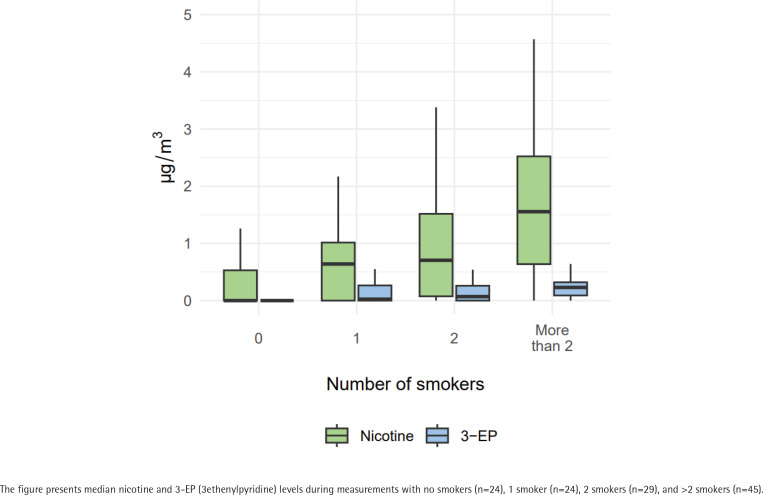
Nicotine and 3-EP levels at public outdoor spaces by number of smokers, the Netherlands, 2021

**Figure 2:**
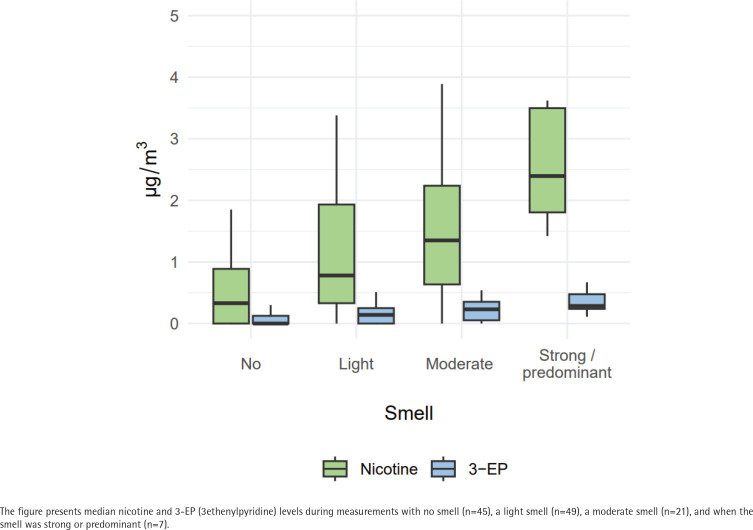
Nicotine and 3-EP levels at public outdoor spaces by tobacco smell intensity, the Netherlands, 2021

We calculated three separate models for predicting 3-EP. The results of these models are presented in Table 3. Mean 3-EP levels were higher when smokers were present than when they were not (B=0.17; 95% CI: 0.08–0.26, p<0.001). Also, mean 3-EP levels were higher when more smokers were present (B=0.02; 95% CI: 0–0.04, p=0.018). Finally, mean 3-EP levels were higher when the smell of tobacco smoke was stronger (B=0.08; 95% CI: 0.03–0.14, p=0.003). Figures 1 and 2 present the median nicotine and 3-EP levels by number of smokers and by level of tobacco smell intensity.

**Table 3:** Mixed linear models for predicting 3-EP levels at public outdoor spaces in the Netherlands in 2021

	*B*	*SE B*	*df*	*t*	*p*
**Condition**					
Constant	0.16	0.32	49	0.50	0.617
Condition	0.17	0.04	95	3.82	**<0.001**
Temperature	-0.01	0.01	38	-1.00	0.325
Humidity	0.00	0.00	48	0.40	0.692
**Number of smokers**					
Constant	0.15	0.33	52	0.44	0.659
Number of smokers	0.02	0.01	112	2.40	**0.018**
Temperature	-0.01	0.01	40	-0.62	0.542
Humidity	0.00	0.00	49	0.60	0.551
**Tobacco smell**					
Constant	0.18	0.33	50	0.54	0.590
Tobacco smell	0.08	0.03	111	3.02	**0.003**
Temperature	-0.01	0.01	40	-0.61	0.545
Humidity	0.00	0.00	47	0.29	0.776

The table shows three separate mixed linear models for condition (no smokers vs smokers, reference: no smokers), number of smokers (range: 0–14), and tobacco smell (range: 1–4). We conducted 5 measurements per location. To control for this, location was added as a random effect. Significance: p<0.05.

## DISCUSSION

This study showed that outdoor smoking causes detectable airborne nicotine and 3-EP levels in public outdoor spaces. We found significantly higher airborne nicotine and 3-EP levels when smokers were present than when they were not. Moreover, regression analyses suggested that nicotine and 3-EP levels were higher when more smokers were present and when the smell of tobacco smoke was stronger. To our knowledge, our current study is one of the first studies to investigate both nicotine and 3-EP levels in outdoor locations. As such, this study showed that both indicators are useful in determining levels of secondhand smoke in various outdoor locations.

### Nicotine and 3-EP levels in public outdoor spaces

Our results are in line with earlier findings from several Spanish studies. We found a median nicotine level of 1.33 μg/m^3^ at all outdoor locations and a median level of 0.48 μg/m^3^ at café terraces specifically. This is similar to López et al.^16^, who found a median nicotine level of 0.66 μg/m^3^ in outdoor settings near restaurants and 0.29 μg/m^3^ near bars. In addition, Fu et al.^12^ registered a median level of 0.54 μg/m^3^ on café terraces. Similar to the findings from the current study in the Netherlands, Fu et al.^12^ found that the number of smokers and the tobacco smell intensity predict airborne nicotine levels. While Fu et al.^12^ used a dichotomous variable for smell (present vs absent), we used a continuous scale for smell intensity. Also, while Fu et al.^12^ used a passive sampling method for collecting nicotine samples, we used an active one using thermal desorption tubes. Since both studies found similar results, both methods appear useful in investigating airborne nicotine levels in outdoor locations.

Similar to nicotine, 3-EP was associated with the observed number of smokers and tobacco smell intensity in our study. In line with previous research^11^, 3-EP levels in our study were much lower than nicotine levels. Still, we found a similar association between 3-EP levels and the presence of smokers and tobacco smell. 3-EP levels were significantly higher when more smokers were present and when the tobacco smell was more intense.

### Smoke-free policies

While an increasing number of outdoor public spaces have become smoke-free in the Netherlands, secondhand smoke exposure remains a cause for concern. This level of exposure is often so high that non-smokers are annoyed by it. A study showed that 40% of non-smokers in the Netherlands feel annoyed by secondhand tobacco smoke outdoors, particularly on outdoor café terraces^17^. This finding suggests that many non-smokers are exposed to considerable levels of tobacco smoke in public outdoor spaces. One way to reduce this exposure is to create more smoke-free outdoor spaces.

This study showed that non-smokers in the Netherlands are exposed to detectable levels of nicotine at the entrances of public buildings, near public transport stops, and on café terraces. While smoking is often banned at such locations in other countries^18,19^, we join earlier calls for more comprehensive outdoor smoking bans at or near those types of locations^1,20,21^.

### Strengths and limitations

A strength of this study is that this research was conducted at various location types across cities in the Netherlands. By not limiting our data collection to a single city, single location, or a single time frame, we were able to collect reliable data on nicotine and 3-EP levels at various types of outdoor locations. Because of this, the results may therefore be generalizable to various other outdoor locations.

A second strength of our study is that we conducted multiple measurements at the same location. This allowed for paired analyses by including a random intercept for location. As a result, we were able to identify differences in nicotine and 3-EP exposure independent of location. By having both one measurement without smokers and four measurements with varying numbers of smokers at the same location, we were able to identify evidence for dose-response relationships between exposure to nicotine and the number of smokers, and exposure to nicotine and tobacco smell intensity. We were also able to conduct the same analyses for 3-EP.

The first limitation of this study was that – due to equipment malfunction – we were unable to use fine particle data in our analyses. Using a SidePak^TM^ AM520 personal aerosol device with an integrated air pump, we measured levels of fine particulate matter (PM_2.5_) in real time. In line with previous research^21^, the monitor was fitted with a 2.5 μm impactor and logged PM_2.5_ concentrations at 1s intervals with a calibration factor of 1.00. Even though the SidePak^TM^ AM520 had been working properly at the beginning of the study, over the course of the data collection there were occasions where we noted higher levels of fine particles than expected. These data suggested a systematic overestimation of background exposure (when no smokers were present). Despite several interim calibration efforts by the researchers, this systematic inaccuracy became only apparent after collecting all the data. As a result, we decided not to report our fine particle data in this article. The nicotine and 3-EP levels presented in this study were unaffected by this, as these variables were not measured using the SidePak^TM^ AM520.

A second limitation was the use of a subjective measure for tobacco smell intensity. Even though we attempted to create a more objective outcome by giving the data-collecting researchers specific instructions for the various levels of smell intensity, this variable remained a highly subjective one. As such, caution is warranted when interpreting this variable and the results. Despite this, results do suggest that there might be an association between the perceived tobacco smell intensity and the level of nicotine one is exposed to in public outdoor locations.

A third limitation might be that this study was conducted during the COVID-19 pandemic. We collected data between May and November 2021. From March 2020 to April 2022, the Netherlands government imposed various coronavirus measures, such as social distancing (1.5 m) and mandatory facemasks on public transport^22^. In addition, hospitality venues were required to impose a minimum distance of 1.5 m between tables and patrons. It is likely that smokers and non-smokers alike were more aware of their physical distance with regard to other people and were more likely to keep a greater distance from others, including distance from members of the research team. Also, as health has become a major concern in society during the COVID-19 pandemic, smokers might have been more aware of their unhealthy behavior and might have felt impelled to reduce their smoking or hide their smoking from others. During our observations at café terraces, we noticed that many smokers kept their lit cigarettes under the table. The results found in this study might therefore be an underestimation of the nicotine levels that may be found under conditions without any coronavirus restrictions.

A final limitation is that we only included two covariates (i.e. temperature and humidity) in our models. Secondhand smoke exposure in public outdoor spaces is dependent on many more variables, such as wind direction, wind speed, the presence of roofing, etc. Due to the limited number of covariates used in this study, the effect of our main predictors on nicotine and 3-EP levels may have been overestimated. Future research may investigate which other factors may also explain outdoor secondhand smoke exposure.

## CONCLUSIONS

This study showed that non-smokers in the Netherlands could be exposed to nicotine and 3-EP when they are near smokers outdoors. The level of nicotine and 3-EP exposure is higher when more smokers are nearby. The data also suggested that the intensity of tobacco smell might also be related to nicotine exposure: the stronger the smell of tobacco smoke, the more nicotine one is likely to be exposed to. Finally, this study showed that both nicotine and 3-EP are useful in determining levels of secondhand smoke in various outdoor locations.

## Supplementary Material



## Data Availability

The data supporting this research are available from the authors on reasonable request.
