# Supplementary material for: Secondhand smoke exposure in public outdoor spaces in the Netherlands: The stronger the smell, the more exposure to nicotine
Source: Tob Induc Dis. 2024 May 17;22:10.18332/tid/186952. doi: 10.18332/tid/186952 (PMC11100306; doi:10.18332/tid/186952)
Supplement: Supplementary file 1 [file TID-22-81-s1.pdf]

Supplementary Material

Table S1. Number of measurements, weather conditions, and flow rate per location (the Netherlands, 2021).

Location code [a]	No smokers	With smokers	Total	Mean humidity (%)	Mean temperature (°C)	Flow rate [b] (cc/min)
**Total**	**25**	**97**	**122**	**72**	**18**	
EN1	1	4	5	70	21	400
EN3	1	4	5	74	13	400
EN4	1	4	5	85	15	400
EN5	1	4	5	77	15	400
EN6	1	4	5	76	13	400
EN7	1	4	5	87	14	400
EN8	1	4	5	88	13	400
PT5	1	4	5	62	21	400
PT6	1	4	5	48	24	400
PT7	2	3	5	91	20	400
PT8	1	4	5	93	13	400
PT9	1	4	5	80	13	400
PT10	1	4	5	96	10	400
PT11	1	4	5	95	12	400
PT12	1	4	5	85	12	400
PT13	1	4	5	88	14	400
CT1	1	4	5	56	17	300
CT2	1	4	5	57	17	300
CT3	1	4	5	-	24	300
CT4	1	5	6	42	26	300
CT6	1	4	5	44	26	400
CT7	0	5	5	47	26	400
CT9	2	3	5	63	21	400
CT12	1	5	6	60	22	400

[a] Location codes for building entrances (EN), public transport stops (PT), and café terraces (CT). [b] Flow rate of the air pump attached to the thermal desorption tubes.

Part 1. Details of the GC-MS settings and analyses.

Chemicals

Standards of the target compounds in analytical grade (purity ≥ 95–99%) were used to optimize identification accuracy. 2-propanol was purchased from Supelco. Nicotine (purity > 99 %) and 4-ethenylpyridine (4-EP purity > 95%) were obtained from Sigma-Aldrich.

Standard solutions and equipment

For the qualification of 3-EP and nicotine, the standards (for 3-EP quantification was 4-EP used) were individually dissolved in 2-propanol. For quantification, eight nicotine and six 4-EP standards ranging 1.5 ng – 300 ng were spiked on the TD tubes. We then analyzed them with a Agilent 7890B gas chromatograph equipped with an Agilent 5977 single-quadrupole mass spectrometer (Agilent Technologies, Amstelveen, the Netherlands) and a multipurpose sampler (MPS-2, Gerstel, Mühlheimander Ruhr, Germany). We used TD tubes with TENAX GR Desorption tube GERSTEL-TDU. These tubed contained a barcode and were preconditioned. We used Da Vinci laboratory solutions Gerstel 020823-005-00. All TD tubes were again conditioned before re-use.

GC–MS conditions

We used an Agilent 7890B GC system with an 5977A single-quadrupole mass spectrometer. They were equipped with a Gerstel MPS auto-sampler and a TDU unit with a quartz wool glass CIS liner. Compounds were chromatographically separated using an Agilent HP-5MS 30m x 0,25 mm, 0.25 µm with helium as a carrier gas in a constant flow rate of 1 mL/min. The temperature program was set at 50 °C (hold for 0 min), then ramp to 260 °C with 10 °C/min (hold for 20 min). The total run time was 41 min. The injector temperature and temperature of the transfer line were set at 250 °C and 280 °C, respectively.

The MS operated in a positive electron impact (EI) mode with an electron energy of 70 eV. The MS quad temperature was set on 150 °C and the MS source was set on 230 °C. After each test sample, a blank TD sample was included to control for carry-over effects.

Qualification of 3-EP and nicotine was performed in Full scan/SIM mode covering 29-250 *m/z*, a range sufficiently broad to cover the analytes. Qualification was based on the retention times and the MS spectra of the individual standards. The response of GC-MS was ultimately linear with the concentrations of nicotine and 3-ethenylpyridine at different ranges. To verify the presence of nicotine and 3-EP in each TD sample, retention times and the mass spectra were confirmed using those of the standards and the NIST. Concentrations were reported with a signal at least 10 times higher than noise.

Table S2. Recovery rates.

Theoretical value	Nicotine		3-EP	
Control (ng)	Measured (ng)	Recovery (%)	Measured (ng)	Recovery (%)
1.5	1.6	106	-	-
4.0	4.2	105	-	-
10.0	9.0	90	-	-
12.5	14.4	115	-	-
20	19.8	99	-	-
35	36.2	104	-	-
50	49.4	99	-	-
300	326.2	109	-	-
12.5	13.6	109	-	-
12.5	13.0	104	-	-
62.5	-	-	60.8	97
131.0	-	-	133.9	102
262.0	-	-	262.8	100
525.0	-	-	531.4	101

Note: The response of the GC/MS analysis was linear with the concentrations of nicotine and 3-ethenylpyridine in different ranges.

© **2024 Bommelé J. et al.**
